# Exploring the acceptability of controlled human infection with SARSCoV2—a public consultation

**DOI:** 10.1186/s12916-020-01670-2

**Published:** 2020-07-07

**Authors:** D. Gbesemete, M. Barker, W. T. Lawrence, D. Watson, H. de Graaf, R. C. Read

**Affiliations:** 1 Faculty of Medicine and Institute for Life Sciences, University of Southampton, School of Clinical and Experimental Sciences, NIHR Clinical Research Facility and NIHR Southampton Biomedical Research Centre, University Hospital Southampton NHS Foundation Trust Mailpoint 218, University Hospital Southampton NHS Foundation Trust Tremona Road, Southampton, SO16 6YD UK; 2 MRC Lifecourse Epidemiology Unit, University of Southampton, D08 Institute of Developmental Science and NIHR Southampton Biomedical Research Centre, University of Southampton and University Hospitals Southampton NHS Foundation Trust, Southampton, UK; 3 School of Human Development and Health, Faculty of Medicine, University of Southampton, D08 Institute of Developmental Science, University Hospitals Southampton NHS Foundation Trust, Southampton, UK; 4 Faculty of Medicine and Institute for Life Sciences, University of Southampton, School of Clinical and Experimental Sciences, NIHR Clinical Research Facility and NIHR Southampton Biomedical Research Centre, University Hospital Southampton NHS Foundation Trust, University of Southampton, Southampton General Hospital, South Academic Block, Mailpoint 814, Tremona Road, Southampton, SO16 6YD UK

**Keywords:** Controlled human infection model, SARSCoV2, COVID-19, Public consultation

## Abstract

Rapid development of an effective vaccine for SARSCoV2 is a global priority. A controlled human infection model (CHIM) would accelerate the efficacy assessment of candidate vaccines. This strategy would require deliberate exposure of volunteers to SARSCoV2 with no currently available treatment and a small but definite risk of serious illness or death. This raises complex questions about the social and ethical acceptability of risk to individuals, given the potential benefit to the wider population, and as such, a study cannot be done without public involvement. We conducted a structured public consultation with 57 individuals aged 20–40 years to understand public attitudes to a CHIM, and pre-requisites for enrolment. The overall response to this strategy was positive, and many would volunteer altruistically. Carefully controlled infection is viewed as safer than natural exposure to wild virus. The prolonged social isolation required for the proposed CHIM is considered an obstacle but not insurmountable, with reasonable compensation and supportive care. Given the significant level of public interest, a CHIM should be done as open science with regular, controlled dissemination of information into the public domain. Importantly, there was a strong view that the final decision whether to conduct a CHIM should be in the hands of qualified and experienced clinician-scientists and the authorities.

## Background

Controlled human infection with pathogens is increasingly used in research and development, despite the obvious ethical issues [1] and regulatory considerations [2]. In the current COVID-19 emergency, if the scientific community were able to use a controlled human infection model (CHIM), it could enable rapid understanding of pathogenesis, induction of immunity and immune mechanisms of resistance to disease. It would provide a test bed for novel diagnostics and treatments, especially between the first and subsequent waves of the pandemic, when natural infection will be relatively rare.

Perhaps the most urgent need at the current time is the rapid development of an effective vaccine. At the time of writing, more than 100 COVID-19 vaccine candidates are at various stages of development, using a wide variety of different platforms, and a few candidates are now entering early phase clinical testing [3]. In normal circumstances, vaccine development is prolonged, averaging over 10 years from start to finish [4]. With unprecedented international scientific and regulatory effort and co-ordination, a COVID-19 vaccine could be available in a much shorter timeframe, but most realistic estimates put this at 18 months [3, 5, 6]. This timeline is in part due to the phase 3 clinical trials needed to prove efficacy, requiring thousands of participants and months of follow-up. The possibility of accelerating this process with the use of a CHIM has been suggested [6, 7].

## How would a controlled infection model help?

A CHIM vaccine-challenge experiment tests the efficacy of a vaccine to prevent infection and/or disease in a small cohort of volunteers. Typically, participants are given a test or control vaccine and 2–4 weeks later are inoculated with the pathogen of interest via the most physiologically relevant route of administration. The number of participants enrolled is calculated to meet a statistically significant and public health-relevant change in clinical or microbiological endpoints, but is usually in the tens rather than the hundreds. This is in contrast to the huge cohorts of participants required in a population-based clinical trial. As multiple putative vaccines emerge over the next few months, as likely they will, a CHIM with SARSCoV2 would facilitate rapid ‘ruling out’ of ineffective vaccines. This alone would prevent wasted time and millions of dollars on large phase II/III studies of ultimately unsuccessful vaccine candidates. This strategy has been successful in evaluating malaria [8], pneumococcal [9] and typhoid [10] vaccines and in providing evidence which contributed to Food and Drug Administration (FDA) approval of a cholera vaccine [11, 12]. It is a powerful technique.

## Should a controlled infection with SARSCoV2 be done?

The ethical issues are complex [7]. Evaluation of any CHIM would take into account the risk, for the individual participant, the study team and the wider community, and potential benefit of the study for society, the recognition and treatment of induced disease, and the quality of care and scientific approach [7, 13]. Of paramount importance is self-determination of participants and rigorous application of informed consent procedures. At its simplest, it can be argued that provided each of these requirements is met and a fully informed volunteer gives consent to infection with a novel pathogen whilst understanding that he/she may come to harm, then a study is ethical. There is an overarching question, however, which is the business of not just the scientific community but also the general public: a clinical study may be ethical, but *should it be done*?

The risk for the individual in a CHIM with SARSCoV2 is not clear. In natural COVID-19 disease, most infections cause only mild to moderate symptoms and the level of asymptomatic infection is unknown. However, an estimated 20% of infections are severe [5] with an infection-mortality rate of approximately 2% in China [14]. The infection-mortality rate varies significantly between countries [15] which is likely due to differences in the availability of testing, as well as population characteristics and available health care resources. Severity and mortality are heavily weighted towards older age groups [5] and those with underlying health conditions. It is estimated that the infection-mortality rate (95% confidence interval) in 20–29-year-olds is 0.0309% (0.0138–0.0923) and in 30–39-year-olds is 0.0844% (0.0408–0.185). In contrast, the estimated infection-mortality rate in the over 60s is 3.28% (1.82–6.18) [16]. Apart from the risk of disease, another important consideration is whether the infection can be treated. A variety of anti-viral drugs and immunomodulators are currently being trialled, but none is currently proven [5]. Much of the mortality is a consequence of immune-driven acute respiratory distress syndrome [17], and whilst prompt general support can mitigate this, there are well-described cases of mortality, even in younger adults despite intensive care [18]. Apart from risk to the individual, any proposed CHIM with SARSCoV2 will require isolation, and probably incarceration in a clinical environment for up to 4 weeks, to prevent harm to the participant and onward transmission to others. The potential mental health consequences of this should not be underestimated.

Given the unprecedented level of public impact and potential interest, it is important to consider the level of public involvement in and ownership of a CHIM. Public consultation has been highlighted as a necessary step in the design of any SARSCoV2 CHIM [7]. As a first step towards understanding the acceptability to the public of a potential fast-track CHIM for COVID-19 vaccine discovery, we conducted a small-scale public consultation using focus groups.

## The consultation

Between 6 and 9 April 2020, we conducted seven online focus group discussions with 57 adults based in the UK and aged between 20 and 40 years. Individuals in this age range were chosen in order to represent those likely to be enrolled in a SARSCoV2 CHIM. Researchers from the National Institute for Health Research (NIHR) Southampton Biomedical Research Centre (BRC) public and patient involvement team identified someone in the target age range who then approached and recruited others in the same age range within their social and work networks. All were sent information electronically giving brief details of the consultation topic and process and were offered a small financial incentive for taking part. Discussants’ characteristics are outlined in Table 1. Focus group discussions were conducted using Zoom Pro and were audio-recorded using Open Broadcast software in order to ensure confidentiality.

**Table 1:** Characteristics of focus group members (*n* = 57)

Women/men	42/15
Age group (years)	
20–25	16
26–30	26
31–35	14
36–40	1
Employment	
Undergraduate student	6
Postgraduate student/early career researcher	7
Professional (office-based)*	31
Nursing	4
Other skilled/semi-skilled**	7
Stay-at-home mum	2
Current location	
Southampton	30
London	7
South East England	11
South West England	3
Midlands	4
Wales	2
Total	57

*Professionals includes sales, marketing, recruitment, teaching, engineering, IT, data analyst, legal secretary. **Other skilled/semi-skilled includes plumber, cabin crew, waitress, nanny, singer

A protocol was outlined to the discussants in which healthy volunteers would be inoculated intra-nasally with the lowest measurable dose of SARSCoV2 and then monitored remotely in self-isolation for up to 28 days. During this period, they would undergo regular medical checks and provide respiratory tract and blood samples. Discussants were told that volunteers might be entirely asymptomatic but were more likely to develop mild or moderate symptoms during this period. Should this happen, they would then be admitted to a hospital clinical research facility and monitored as an inpatient with further tests and 24-h nursing. It was made clear that, in the event of illness, the clinical investigators would be unable to predict exactly the likelihood of development of severe symptoms and death. Specifically, it was suggested that the risk of death was in the range 1:100 to 1:10,000. The discussants were told that payment for taking part in the CHIM would be in line with other recent CHIMs requiring an inpatient stay, amounting to approximately £1000 per week. It was suggested that given the complex ethical issues and the potential public interest in such a study, it would be important to have complete transparency with the scientific community and the public. The study might therefore be conducted as ‘open science’ in which the public were fully informed prior to the study taking place and then able to follow in real time the progress of the study. The groups were then guided through a discussion by an experienced facilitator, supported by an observer, using a semi-structured discussion guide. This was reviewed after each focus group by the research team and revised when it was felt topics needed further exploration. Notes taken and recordings of the discussions were synthesised to address the following questions. Illustrative direct quotes from discussants are provided in Table 2*.*

**Table 2:** Illustrative quotes

Focus group	Quote
*Question 1: Should this study be done?*	
FG1	I’m able to work from home, but I still feel quite helpless and I think there is quite a lot of people who would want to do something … I think it’s something that I would be interested in.
FG2	Emphasis on this being a ground-breaking piece of research and all the people you could help, loads of emphasis on that.
FG6	The scientists who know what they are talking about make the decision to put forward the study.
FG6	If I knew that it’s something been robustly approved by an ethics board, I’d be fine.
FG7	I do not think the public should be the people to decide whether it goes ahead or not, it’s for higher up I think.
*Question 2: What might volunteers be particularly concerned about?*	
FG3	I think I feel really anxious given what you are seeing in media everyday about the number of people who are dying and the young people that are being really unwell with it.
FG1	I think at this point most people have accepted that most people will probably get it in the upcoming months, and I would much rather have it in a controlled environment in a hospital than be at home in isolation. And obviously if something does go wrong, they have doctors around them to care for them.
FG1	I live with my family, like worried that I was going to pass something on to them so at least we would be in a controlled environment, I would be away from them and they would not get infected.
FG4	I’m pretty sure that actually my mum would not let me take part in this even though I’m 32 years old. I would not have parental consent!
FG2	I’d like to know that my employer would support me in being a part of this process.
FG5	Is there enough space, if so, are they not taking the (hospital) beds away from the people that have got ill and have not actually voluntarily got the disease.
FG5	I guess like those things we take for granted such as internet access, books, Netflix, but I think also just having the ability to have a bit of fresh air … I think your mental health would be seriously impacted stuck inside all day.
FG6	You could have calls together so you can talk about the experience together to support each other mentally, psychologically.
FG6	You could possibly have a psychologist to support how people might be feeling.
*Question 3: How best can we communicate risk to potential volunteers?*	
FG1	I would just be a little apprehensive and worried about maybe the cohort of 20–40, young people think that they are actually a bit invincible, and actually we know that the risk in unknown.
FG7	I’m quite numbers driven, so I’d want to know … what are the chances of me going into intensive care … or dying.
FG2	The fact that you are being so open and honest about the fact that this is not zero risk.
FG3	1 in 100 or 1 in 10,000, if you could make it more relatable, maybe the chances are of being in a plane crash or getting hit by a bus.
FG4	Be able to ask questions face-to-face with somebody, I would not just want a long information sheet.
FG3	Maybe it’s worth gathering the positive data, how many people have had this and shown little symptoms or no symptoms and got through it and talk about the positive numbers of the effects.
FG4	I think I would want to know if they are for everyone or if there were different numbers for my age group.
*Question 4: How much financial compensation is acceptable?*	
FG1	As a covering of a loss of earnings, this feels about right and it is necessary to do that because there is risk.
FG6	I’d be like WOW that’s almost double my earnings so I’d be like yes sign me up … but then I would worry on the opposite side about who would that attract.
FG7	My only moral concern is … you wonder if people will be doing it for the wrong reason … you want people to do this study knowing what they are going into … if they are sitting there with dollar signs in their eyes they might not listen to all the other things … they do not hear the rest of what you say, they do not hear any of the risks.
FG4	Could it be just that when you introduce the money aspect of it, actually when you are advertising for participants … it’s not until someone has interest and they have consultation with someone that then they find out.
*Question 5: What do you think about details of this study being made available to the public?*	
FG2	It would be perceived in quite a positive light, that you were doing something to help the nation recover from this epidemic a lot quicker, so personally I think I see it as a more positive thing, like look at these people they are doing something really great … helping us come out of lockdown quicker.
FG1	I think actually seeing the symptoms or the progress that people are making across the weeks might be more useful to more people than a number going up weekly.
FG6	People share their symptoms … I’ve definitely benefited from those videos where people have actually stated what they feel ... they are the most real stories I’ve heard so far.
FG6	Making sure that it’s clear at the beginning that this is going to be out in the media and if they want to be a spokesperson for it, record their time and update people then they have the option to but absolutely have no pressure to.
FG5	The negative people would be the people that go against vaccines in general which seems to be something that I keep seeing all over everywhere at the moment. And that I suppose is one of the reasons I would not want my name out there just because there are people out there … some of them are quite cruel online.
FG7	I’m sure we are all familiar with how badly the media reports anything to do with any scientific study … if you can potentially control the dissemination of that material and people have got such an interest, so in terms of keeping that message on track that could be quite useful.
FG7	I live in *** and it’s a very small place and everyone knows everyone, and I think if they found out I was taking part they’d all start putting red crosses on my driveway and telling my house is infected, and I just do not think that would really go down very well … they would not be very impressed potentially bringing something back to the area.
FG7	The last thing you want to do is turn it into some really sick version of *Love Island*.
FG7	Conspiracy theorists are going to have a field day if they find out that a study is deliberately infecting people and they get the wrong end of the facts, but they are having a field day anyway it should not stop something important going ahead.

### Question 1: Should this study be done?

The first question addressed the acceptability of such a study taking place. Importantly, no one felt that the study should not be done. On the whole, people were positive about the study and the scientific effort being made towards ending the pandemic. Many said they would be prepared to take the risk and volunteer for altruistic reasons. They wanted to help with finding a vaccine. When asked who should be responsible for deciding whether or not to run such a study, discussants felt that the level of knowledge and understanding required meant that it should be the scientific community and ethics committees who made a final decision, rather than the general public.

### Question 2: What might volunteers be particularly concerned about?

The second question was used to explore specific concerns and constraints that might impact an individual’s decision to participate. Several people expressed significant personal anxiety about the unknown risks of deliberate infection and experiencing severe symptoms given no known treatment. This anxiety was mitigated in some instances for those who understood that they were highly likely to get infected by natural exposure anyway so felt they might benefit if the infection was tightly controlled and monitored. Many expressed concerns about taking away beds, ventilators and nursing care from those infected naturally, and would want reassurances that this was accommodated for by the trial team. There was clear concern about potential onward transmission to family or other household members. The majority felt that people with dependents or who lived with others should be excluded from the study or spend the entire isolation period in hospital. Many said they would have to consult with family and friends before agreeing to take part, and some felt they would need to include their employers in their decision.

Volunteers’ mental health, including anxiety about the disease as well as existing mental health problems, was mentioned several times. One suggestion was that a psychologist should be employed to support volunteers throughout the study. Another was that volunteers should be networked so that they could support each other online. Isolation away from home in a hotel setting was an attractive prospect for a number of them. Volunteering for the study was felt to be particularly appealing at the moment during the current lockdown period, since the study conditions would not be very different from what many were currently experiencing. If this were to extend past the lockdown and isolation period, it would become less attractive to volunteers, exceptions to this being those who have become unemployed during the lockdown period.

### Question 3: How best can we communicate risk to potential volunteers?

The unknown risk involved in participation in such a study is an important consideration. This question addressed that uncertainty and how best to communicate it to potential participants. There was some concern about young people volunteering because they felt invincible and blasé about their relatively low risk of infection. For those who understood the risk and were still prepared to take part, they said that they would want to be convinced about the benefits of participation. Risk statistics were not considered helpful to most people in understanding what sort of a risk they would be taking. They suggested that communications about risk should be brief and simply expressed using tangible comparisons, e.g. the risk of being a tree surgeon or being hit by a bus. Openness and honesty about the risks associated with the trial were welcomed and found to be reassuring, even if it was made clear that the exact size of the risk was unknown. Discussants wondered if it would be possible to represent the numbers of people who get mild infection or survived serious infection and thus convey a more balanced picture of the risk. They also suggested that the risks of severe infection or death be stratified by age group, so that they could make a judgement based on data for their own age.

### Question 4: How much financial compensation is acceptable?

Discussants’ views were sought about the suggested level of financial compensation. They were asked to consider whether it would provide sufficient reimbursement for the time and inconvenience of participation and the necessary self-isolation, without causing undue inducement to individuals who might be suffering financially as a result of the pandemic. This was met with varying responses. For some, the weekly rate of £1000 was considerably more than a week’s wages and would be acceptable. For others, this was not enough to compensate for the risk of complications resulting from being infected. Some felt this was enough money to act as an incentive to take part for young people many of whom earn low wages or who are currently out of work, and that the public might be concerned about this. The suggestion was that the amount of compensation not be discussed until volunteers had already expressed interest and understood the risks.

### Question 5: What do you think about details of this study being made available to the public?

Discussants were asked about the concept of an ‘open science’ study and what level of information should be shared with the public about the study and individual participants, and in what way. Everyone thought that news that such a study was to take place should be made public. Many felt that daily or weekly announcements should be made about the progress of the study and about how the volunteers were faring. Opinions varied about whether people should be identified. It was suggested that volunteers should be offered the option of remaining anonymous during the study. One potential scenario was that one or two volunteers could become the public face of the study and would be involved in blogging and vlogging about the study and posting their progress on social media. Some felt it would be helpful to the public to be able to read ‘real’ stories from people as their symptoms progressed as a way of educating the public about disease symptoms. Strong reservations were expressed about the level of information to be made available to the public about individual volunteers. Some felt it would stigmatise volunteers if they let it be known that they had been infected. Others thought it would lay them open to ‘trolling’ and abuse from groups opposed to vaccines. Important points were made about the need to handle with care and skill the publicising of information about this study. The extraordinary amount of public interest and press coverage of the pandemic, much of it terrifying, would make this a difficult job. It was felt that publicising the study from the outset would allow the scientists to take control of the dissemination of information and findings. It is clear that if this study were to go ahead in the public eye, a skilled public relations firm would need to be employed to manage the process.

Overall recommendations for the design of recruitment materials are given in Table 3*.*

**Table 3:** Recommendations for recruitment

This public consultation suggests that recruitment materials for this study could usefully include the following:	
Young healthy adults have the lowest risk of severe illness.	
The risks of severe illness and death are not known with any certainty.	
Being infected under controlled conditions with immediate access to specialist health care may reduce the risk of disease progression.	
Having underlying health conditions, living with dependents or being key workers means they will not be considered.	
Clear explanation that the study would require a period of strict isolation of up to 28 days.	
Offering the choice of whether they spend time at home in isolation with access to on-demand hospital facilities or whether they isolate for the whole period in hospital or a dedicated hotel/private accommodation.	
Availability of research team members to discuss their involvement and answer questions about the study from volunteers, their families and their employers.	
Availability of a psychologist to support volunteers through their isolation.	
All needs for food, transport, pet care, etc. taken care of and high-quality internet access whilst in isolation.	
Cover for loss of earnings whilst in isolation or ill would be offered.	

The overall response to the public consultation was positive. One discussant said:

I do think it would be really positive, I think it would be really, really beneficial if something like this did go ahead. I just think it would be a credit to everyone who is involved … thank you for even thinking of it as it’s really difficult to put this out there and to just even get general feedback and for people to be willing to even contemplate it, and ask these really hard questions. FG6

## Discussion

From an investigator’s perspective, the inability to quantify the risk to participants would normally render a CHIM inadvisable. Considering the risks to participants and the potential benefits to the population, a CHIM with SARSCoV2 could not be performed without public consultation. This rapid small-scale public consultation has provided insight into the public view of such a CHIM taking place, and some of the important considerations in study design.

The views obtained in this consultation were limited to individuals in the UK and those in the age range 20–40 years. This age range was chosen to represent the views of those likely to be eligible for such a study. There is also a significant preponderance of women and a high proportion of discussants who were either professional or in higher or postgraduate education. This consultation was not, however, designed to produce views that are representative of the UK population as a whole, or of those from different countries or cultures. Our intention in running these discussion groups was not to provide a representative sample of views of the UK public so much as to gain a nuanced understanding of the perceptions of a range of people who would be the target group for such a study. The purpose was to understand how likely young adults were to volunteer to take part in a CHIM and features of the study that might make it more or less likely that they take part. That most of the participants were women is likely to have coloured the findings from the consultation, but it is not possible without carrying out more consultations with men to know in what way the findings are different. The fact that many of the participants were professional reflects the dominant employment in the area of the UKs from which participants were recruited. Further public consultation may be beneficial in revealing different views among a broader range of individuals if such a study is to take place.

## Conclusion

This public consultation has revealed the public to be largely positive, despite the unknown but possibly considerable risk, with no discussants expressing a view that it should not be done. When considering their own involvement as a volunteer, many expressed concerns about the personal risk, or the impact on, or risk to, family members or friends. Some stated that they would be unable to be involved due to their personal circumstances, but overall, a significant number would consider participation with the altruistic motivation of contributing to the greater good. It seems likely that it would be possible to enrol sufficient volunteers for such a study.

If a CHIM were to proceed, the complexities of the methodological and governance systems required to make the study as safe as possible are daunting. Investigators are rightly fearful of harming a volunteer. Apart from the deep remorse that would be felt, the reputational risk to the scientific field of controlled infection in the event of an accident or rare complication is considerable. Important considerations for the consent process have been highlighted. It is important to be as open and honest as possible about our lack of precision regarding the risks of symptoms, serious disease, long-term effects and death. Risks, and the level of uncertainty, must be expressed clearly and tangibly. The involvement of a psychologist in both the consent process and throughout the period of isolation would be essential.

It was agreed that the level of public interest in this study would be high. All discussants felt that it would be important to inform the general public about the study and provide regular updates, both in order to maintain transparency and accountability and to ensure that public information about the study was accurate. However, crucially, the decision about whether or not the study should take place was felt to be one that the scientific community and ethical boards should make, rather than the public. This reveals the level of trust that the public has in medical research, but puts the burden of responsibility on the scientific community. For us, the investigators, this means that we must consult widely and engage with international qualified scientific colleagues before embarking on a CHIM. Such a study with SARSCoV2 will be complex, but possible, and it seems that the public would be supportive. In our view, the scientific community should seek to fund, design and set-up a study as quickly as possible, using an open science model to maximise public involvement, whilst also ensuring there is supportive consensus from scientific colleagues.

## Data Availability

Not applicable

## Abbreviations

BRC: Biomedical Research Centre
CHIM: Controlled human infection model
FDA: Food and Drug Administration
FG: Focus group
NIHR: National Institute for Health Research
UK: United Kingdom
